# Blue period - features of senescence 30 years after beta-galactosidase

**DOI:** 10.18632/aging.206380

**Published:** 2026-05-15

**Authors:** Chisaka Kuehnemann, Christopher D. Wiley

**Affiliations:** 1 Jean Mayer USDA Human Nutrition Research Center on Aging at Tufts University, Boston, MA 02111, USA; 2 Department of Medicine, School of Medicine, Tufts University, Boston, MA 02111, USA

**Keywords:** senescence, aging, biomarkers, SASP, cell death

## Abstract

Cellular senescence is a multi-phenotypic stress or damage response characterized by a stable cell cycle arrest and the secretion of a myriad of biologically active molecules, commonly known as the senescence-associated secretory phenotype (SASP). Thirty years ago, the identification of activated beta-galactosidase during senescence led to one of the first characterizations of senescent cell accumulation during biological aging. Since then, interventions that either selectively eliminate senescent cells or suppress the SASP have demonstrated that they are a major etiological agent for several degenerative pathologies associated with aging. As interest in the development of targeted therapeutics for senescence has grown, so too has the interest in the study of these cells. This has resulted in discovery of new features and phenotypes that often associate with senescence. Here, we review several of these key features of senescence, highlighting the strengths and caveats associated with each.

## INTRODUCTION

The origins of senescence lie in the refutation of reports from Nobel laureate Alexis Carrel. By serially culturing chicken fibroblasts in media supplemented with chicken serum, Carrel described a state of cellular immortality by which cells could proliferate indefinitely [1]. It has since been suspected that the addition of sera may have inadvertently contaminated the cultures with new fibroblasts [2, 3]. In the 1960’s, Leonard Hayflick published seminal manuscripts demonstrating that primary human fibroblasts do not have unlimited replicative potential, contrary to Carrel’s findings, and in fact undergo a state of permanent arrest in response to serial cultivation [4, 5]. This phenomenon, now known as cellular senescence, was highly controversial at the time. Indeed, Hayflick’s initial submission to the Journal of Experimental Medicine was rejected, with the rejection letter signed by future Nobel laureate Peyton Rous - who not only cited the common belief on the immortality of cultured cells, but described Hayflick’s speculation of this divisional limit as a driver of aging as “rash”. In the years since this first characterization, senescence has expanded from a perceived tissue culture artifact to a *bona fide* driver of aging and degenerative disease.

Discovery of cellular senescence in tissue culture led to speculation of its role in the context of physiology. While the name “senescence” suggested a role in aging, without evidence of increased senescence with age, it remained largely speculative whether senescence was anything other than a tissue culture phenomenon. This changed in 1995, when accidental incubation of x-gal with senescent cells in an acidic buffer resulted in robust blue staining [6]. This senescence-associated beta-galactosidase (SA-β-gal) is common to multiple forms of senescence, and staining of aged human tissues confirmed the increased presence of senescence with age [6]. Several additional features of senescence have been discovered in the thirty years since the description of SA-β-gal, each with their own strengths and weaknesses both in culture and *in vivo* (Table 1). Here we review several of these key features of senescence and how our understanding has evolved over time, noting that there have been both breakthroughs and caveats in the discovery and characterization of these features in the intervening years.

**Table 1:** Key features of cellular senescence and biomarkers.

**Feature**	**Description**	**Markers**
Proliferative arrest	Stable, typically irreversible halt in cell division. Increased cell size and flattened morphology.	Loss of Ki67/PCNA, reduced BrdU/EdU incorporation, increased CDKNs (p16, p21, p27, p15).
SA-β-gal and lysosomal glycosidases	Increased lysosomal β-galactosidase activity detectable at pH 6.0, also increased α-fucosidase.	GLB1, FUCA1, β-glucuronidase.
Senescence-Associated Secretory Phenotype (SASP)	Secretion of inflammatory cytokines, chemokines, growth factors, proteases, EVs, and oxylipins.	NF-κB–driven cytokines, type I interferon response, cGAS–STING activation, EVs, oxylipins (e.g., dihomo-15d-PGJ2).
Nuclear alterations	Structural and epigenetic changes including loss of nuclear integrity and persistent DNA damage.	Loss of Lamin B1 and HMGB1/ 2, cytosolic chromatin fragments (CCFs), DNA-SCARS, TAF, SAHF, SADS.
Mitochondrial dysfunction	Mitochondrial DNA depletion or loss of electron transport chain activity.	Increased mitochondrial mass, ROS, mtDNA release, altered NAD^+^/NADH ratio, ETC dysfunction.
Lysosomal alterations	Increased lysosomal content, altered pH, leakage, and elevated autophagy/exocytosis.	Lysosomal expansion, cholesterol partitioning, autophagy upregulation, lysosomal processing of CCFs.
Metal accumulation	Accumulation of ferrous and ferric iron, copper, zinc, manganese, magnesium, and potassium.	Ferritin/hemosiderin, labile iron; copper transport dysregulation.
Lipofuscin accumulation	Build-up of oxidized proteins, lipids, and metals due to impaired lysosomal degradation.	Autofluorescent granules, Sudan Black B staining.
Persistent cell survival	Persistent cell survival in the presence of cell death stress.	Upregulation of BCL-2 family proteins, decoy receptors, altered DR5/cFLIP. Resistance or sensitivity to necroptosis, ferroptosis, parthanatos.

## Proliferative arrest

From the earliest days of Hayflick and Moorhead, serial cultivation of non-transformed primary cells has been associated with eventual replicative exhaustion and stable proliferative arrest, typically in the G1 phase of the cell cycle [4]. Subsequently, this arrested state now known as cellular senescence was demonstrated in many cell types, and in response to multiple inducers including - but not limited to - telomere attrition [7], genotoxic stress [8], oncogene activation [9], oxidative stress [10], mitochondrial dysfunction [11], or pharmacological inhibition of proliferative pathways [12–14]. Distinct from quiescent cells and other forms of terminal arrest, cultured senescent cells often continue to increase in mass and diameter in the absence of cell division, at least at low cell densities, and this cellular hypertrophy itself may contribute to the cell cycle arrest [15]. The senescence arrest is often referred to as “growth arrest”, but to disambiguate between this increase in size (which occurs in senescence and thus could be considered “growth”) and the arrest of cell division, we will refer to this phenotype as “proliferative arrest” in this article.

Senescence-associated proliferative arrest is commonly detected by a combination of loss of proliferation markers, coupled to elevation of one or more cyclin-dependent kinase inhibitors (CDKNs). In culture, proliferation can be measured by cell counts, markers such as Ki67 and proliferating cell nuclear antigen (PCNA), or incorporation of labeled thymidine analogs (due to thymidine’s preferential incorporation into DNA during replication). Initially, radiolabeled thymidine was used to detect cell cycle arrest, but development of non-radiolabeled alternatives such as bromodeoxyuridine (BrdU; which allows detection by immunofluorescent detection) or 5-ethynyl-2'-deoxyuridine (EdU; which allows detection using CLICK chemistry) have become the preferred approach in more recent years. Each of these markers are lost from senescent cells, and often expressed as percentages of the whole population [16, 17].

Because the proliferative arrest is driven by elevation of one or more cyclin-dependent kinase inhibitors (CDKNs), they can be considered markers of senescence. The two most commonly studied CDKNs associated with senescence are p21 (*CDKN1A/p21^WAF1^*) and p16 (*p16^INK4a^,* one of many transcripts from the *CDKN2A* locus), but two other CDKNs, p27 (*CDKN1B/p27^KIP1^*) and p15 (*CDKN2B/p15^INK4b^*), have also been linked to senescence. All four of these transcripts have been shown to increase with age in at least some tissues [18–21]. Genetic models that eliminate cells with high p21 or and p16 expression have demonstrated a role for both in the promotion of age-related diseases [22–24], but the roles of or p27 and p15 in aging and other senescence-associated conditions remain unclear.

Importantly, cell cycle arrest and CDKNs on their own are not sufficient to identify senescent cells. *CDKN1A* has multiple transcripts with identical ORFs, and the mouse expresses both an abundant transcript that responds to circadian rhythms and genotoxic stress, and a less abundant transcript that increases with age [18] – which cannot be distinguished by standard 3’ RNA-seq techniques. The human gene has a similar set of transcripts, but it is not currently known how these might vary with senescence and aging. Similarly*, p16* is only one of multiple transcripts that originate from the *CDKN2A* locus, including *p19^ARF^* (mouse)/*p14^ARF^* (human), most with a common 3’ end, similar to the *CDKN1A* locus. These shared 3’ ends require alternative methods to detect specific transcripts, such as selective primers, 5’ RNA-seq, or extended reads. In addition, p16 is not only elevated during senescence, but also during some forms of macrophage activation [25–27] and in response to mutation of the retinoblastoma (*Rb*) gene [28]. Both p21 and p27 are also elevated during quiescence [29], and p15 is a potentially important regulator of myeloid differentiation [30, 31]. Finally, CDKNs can be transiently elevated in response to stress or damage, but decline once these are resolved [32]. Thus, CDKNs have roles outside of senescence that cannot be overlooked when studying the senescent phenotype and when considering the effects of CDKN targeting on health outcomes.

Similarly, cell cycle arrest can originate from cellular states other than senescence. Many differentiated cell types - such as neurons, muscle cells, or podocytes - undergo stable cell cycle arrest upon differentiation without being considered senescent. In addition, many cell types undergo G0 arrest, or quiescence, in response to nutrient or growth factors withdrawal, contact inhibition, or from cues from the tissue microenvironment [29, 33], and quiescent cells are often able to resume proliferation in response to changes in these factors.

Finally, there are a growing number of reported exceptions to the stability of cell cycle arrest in response to a variety of stimuli [34–39]. This reversion can extend beyond cell cycle arrest and into other features of senescence [37], but can also result in pro-carcinogenic states [40–43]. These cells with reversible senescence features may be largely indistinguishable from irreversible senescence arrest *in vivo*, so it is unclear how prevalent the persistent cell cycle arrest is in an organism. Thus, while proliferative arrest is a defining feature of senescence, on its own it is insufficient to identify senescent cells.

## Beta-galactosidase and other lysosomal glycosidases

As described above, senescent cells often stain positive for beta-galactosidase. Following the discovery of SA-β-gal, it was subsequently determined that senescence-associated beta-galactosidase was lysosomal beta-galactosidase, encoded by the gene *GLB1,* explaining the low-pH specificity [44]. Surprisingly, it remains somewhat unclear how senescent cells stain positive for SA-β-gal. Western blots suggest that GLB1 protein level is increased during senescence in human [44] and mouse [45] cells, and both *GLB1* RNA and protein levels have been reported to increase with age in mice, where they predict lifespan and health status [45]. By comparison, RNA levels of GLB1 do not appear to increase substantially in cultured senescent human cells, despite the increase in activity [46, 47]. Notably, a recent study suggests that lysosomes may undergo leakage during senescence, and this may explain why SA-β-gal does not appear to exclusively stain lysosomes in senescent cells, though this remains an open question [48].

Another lysosomal glycosidase was subsequently identified as a biomarker of senescence. Alpha-fucosidase (gene: *FUCA1*) is involved in *N*-glycan degradation, releasing fucose in a manner similar to beta-galactosidase does for galactose [46]. Notably, FUCA1 RNA, protein, and activity levels all increase with senescence, and generally more highly than beta-galactosidase – though it is much less-studied than beta-galactosidase at this point. The same report also indicated a small increase in beta-glucuronidase, another lysosomal glycosidase.

Detection of either beta-galactosidase or alpha-fucosidase is subject to a number of confounding variables. For example, either enzyme might be elevated in response to lysosomal expansion or in cell types with high lysosome numbers, regardless of senescent state. For example, macrophages were shown to reversibly elevate beta-galactosidase in response to the secretions of senescent cells [26]. Notably, high confluence can also result in elevated beta-galactosidase staining [6] and *GLB1* expression [29]. Finally, beta-galactosidase positivity can be subjective, and even objective measures (such as intensity thresholding) can reveal SA-β-gal negative cells in cultures that are entirely arrested. Lysosomal enzymes such as SA-β-gal and α-fucosidase often manifest along a continuum rather than as an all-or-none signal, creating a gradient in which higher activity reflects a greater likelihood of senescence. Thus, while senescence-associated beta-galactosidase remains a key feature of senescence, the number of exceptions to this, coupled to newer biomarkers of senescence, suggest that it may no longer be the gold standard it once was.

## The Senescence-Associated Secretory Phenotype (SASP)

Initially, senescence was thought to be a driver of aging through the replicative arrest, which would prevent the regeneration or renewal of a tissue [49, 50], but eventually it became clear that senescent cells secrete a number of biologically active molecules that can have potent local and systemic effects. This senescence-associated secretory phenotype (SASP) was hinted at in findings that unidentified factors found in conditioned media from senescent cells can drive cell non-autonomous effects on wild type and cancer epithelial cells [51]. In 2008, a series of manuscripts identified a number of inflammatory cytokines and chemokines, growth factors, and matrix metalloproteinases as key molecules secreted by senescent cells [52–54]. Since the initial characterization of the SASP, >1000 proteins have been identified as secreted from senescent cells, many of which are inflammatory cytokines driven by either NF-κB and/or in response to a type I interferon response [55–58]. This interferon response is due to activation of the cGAS-STING signaling pathway in response to cytosolic DNA. The source of cytosolic DNA can be variable, as reported sources include de-repression of LINE-1 elements [56], nuclear blebbing of chromatin into the cytosol [57], and release of mitochondrial DNA due to sub-apoptotic minority mitochondrial outer membrane permeabilization [58]. Through these mechanisms, senescent cells can drive sterile inflammation. This makes them a major candidate for driving age-related inflammation, or “inflammaging” [59]. Beyond cytokines, senescent cells also release several molecules associated with vasoconstriction, coagulation, and fibrosis – suggesting a role for senescence in tissue remodeling responses following injury [60–62]. At least some of these factors do not appear to be linked to the inflammatory cytokine production, as the hemostatic features of senescence are activated in the absence of inflammatory cytokine production [62].

Not all SASP components are secreted as soluble proteins. Senescent cells also release extracellular vesicles (EVs) that can have various effects [63–67]. Notably, many proteins found in EVs from senescent cells correspond to similar secreted pathways, including inflammation, extracellular matrix regulation, and hemostasis, whereas the lipids found in these EVs primarily consist of membrane phospholipids and sphingomyelins [67]. Finally, microRNAs can be encapsulated in EVs from senescent cells [68, 69]. Surprisingly, a cell surface protein, DPP4, limits the uptake of these EVs by proliferating cells [63]. Conversely, senescence-associated EVs can transfer senescence signatures from senescent chondrocytes to non-senescent chondrocytes [64] and fibroblasts [66]. In addition to the hundreds of proteins that compose the SASP, several lipid species are released by senescent cells, most notably many oxygenated polyunsaturated fatty acids collectively known as oxylipins [70, 71]. Senescence-associated oxylipins include a combination of prostaglandins, thromboxanes, and leukotrienes that are functionally associated with inflammatory, coagulation, vasoconstriction, algesia, and fibrosis. Furthermore, oxylipin biosynthesis can reinforce senescence, promote proinflammatory features of the SASP, and drive fibrosis. One oxylipin in particular, dihomo-15d-PGJ2, accumulates in the intracellular space and can be detected during senolysis [70]. Thus, multiple factors beyond proteins may play a role in senescence.

As an important caveat, sources of SASP factors are not limited to senescent cells. Many factors, such as viral or bacterial infection, can result in cytosolic DNA and activation of interferon responses independent of senescence. Similarly, inflammatory responses can occur during immune system activation in the absence of senescence, and HMGB1 – released from the nuclei of senescent cells [72], is also released following lytic cell death [73]. With >1000 identified factors comprising at least some version of the SASP [52, 60, 62, 67, 70, 74–76], many different secreted factors might present like the SASP in datasets without originating from senescent cells. Advances in single cell technology have added clarity to the field by allowing identification of senescent cell types and distinguishing between senescent and immune cells in a way that whole tissue/population assays cannot [77–79]. Finally, it is important to note that the SASP is not a single phenomenon, but a collection of phenotypes that can vary with time, cell type, and inducer [11, 52, 60, 71, 80].

## Organelle alterations

### Nuclear changes

Senescent cells undergo several changes to their nuclear architecture that are essential for both proliferative arrest and SASP. Notably, senescent cells lose RNA expression and protein levels of lamin B1 (LMNB1), resulting in structural changes to the nuclear envelope, which results in release of cytosolic chromatin fragments (CCFs) and activation of the cGAS-STING pathway, as described above [57, 81]. Similarly, epigenetic changes during long term senescence can result in LINE-1 element mobilization and activation of cGAS [56]. As LINE-1 element transposition can result in new double-stranded breaks [82], this may also help to reinforce cell cycle arrest. Furthermore, loss of nuclear localization/expression of high mobility group box proteins (HMGB1 and HMGB2) alter nuclear architecture and absence of these factors from nuclei can thus act as biomarkers of senescence [72, 83]. Importantly, many of these markers are reduced during senescence, and as senescent cells comprise a small proportion of cells, even in old age, they cannot be reliably detected in whole tissue assays. Thus, these markers instead require individual cell approaches when analyzing them in tissues.

Nuclear changes can also occur in response to specific inducers of senescence. In response to DNA double strand breaks, telomere attrition, and related stressors, senescent cells develop persistent foci that stain positive for several markers of DNA damage, most notably γ-H2AX and 53BP1. These persistent foci, sometimes called DNA-SCARS (segments of chromatin alteration reinforcing senescence), result in continuous DNA damage signaling – leading to stable cell cycle arrest and promoting parts of the SASP [84, 85]. While initially hinted at in cells with eroded telomeres due to end replication failure, many of these foci can occur at telomeric DNA, resulting in co-staining between telomeres and DNA damage markers - often called telomere-associated foci (TAF). TAF not only occur during replicative exhaustion, but also often in response to other inducers [86]. Because telomeric DNA damage is more difficult to repair, these foci tend to persist and may be a major reinforcer of the cell cycle arrest [87]. Similarly, in response to activation of p16, a subset of senescent cells can form regions of facultative heterochromatin compaction. These senescence-associated heterochromatin foci (SAHF) occur near clusters of proliferation-associated genes and may thus help reinforce senescence [88]. Conversely, satellite heterochromatin can become reactivated, resulting in senescence-associated decondensation of satellites (SADS) [89]. Thus, nuclear changes can include structural, epigenetic, and genomic components.

### Mitochondrial changes

Senescence is often coupled to a loss of mitochondrial function, including increased mitochondrial DNA, mitochondrial mass, and reactive oxygen species [90–92]. In addition, as mentioned above, sub-apoptotic minority mitochondrial outer membrane permeabilization can result in release of mitochondria DNA into the cytosol, driving inflammatory components of the SASP [58]. Mitochondrial dysfunction, including partial or complete mitochondrial DNA depletion or loss of electron transport chain activity, can also drive senescence without the inflammatory SASP components by lowering the cytosolic NAD+/NADH ratio [11]. Thus, loss of mitochondrial function is not only a feature of senescence, but can also influence senescence and the SASP in multiple ways [91, 92].

### Lysosomal alterations

As mentioned above, lysosomal function is altered during senescence, including increased lysosomal numbers and function, leakage, levels of glycosidases, and cholesterol partitioning [46, 48, 93], and decreased lysosomal pH. Macroautophagy and chaperone-mediated autophagy are also elevated during senescence, with increased exocytosis of lysosomal components to the extracellular space, forming a subset of SASP factors [76]. Similarly, CCFs that bud off of the nucleus are processed by the lysosome [94]. This would suggest that dysregulated lysosomal processes are a key feature of senescence, but the relationship between lysosomes and senescence is just beginning to be understood *in vivo* [95]. For example, blocking autophagy can result in buildup of damaged macromolecules and activate signaling pathways that can trigger senescence [96], whereas overexpression of TFEB which controls lysosome expansion - prevents senescence in various models [97]. The role of lysosomes in aging and cellular senescence has been reviewed in more detail elsewhere [95].

## Metal accumulation

A growing body of evidence indicates that senescent cells accumulate elevated levels of several metals, including iron, potassium, magnesium, manganese, zinc, and copper [98–103]. Of these, iron is perhaps the best-studied, with accumulation of both ferrous and ferric iron observed in senescent cells [100, 102]. Hemolysis can drive senescence in nearby cells at least in part by increasing iron, and chelation of iron can lower levels of multiple SASP factors [102]. Iron, stored in hemosiderin and ferritin, can be detected in senescent cells histologically [100, 102], and senescent cells can even be detected by MRI using iron as a target [102]. More recently, copper accumulation in senescent hepatocytes was identified as a vulnerability for their elimination in a mouse model of hepatosteatotic disease [104]. Less is known about the accumulation of other metals, or their consequences, but these may eventually become key markers of senescence as the mechanisms behind their accumulation are elucidated.

## Lipofuscin

Lipofuscin is an intracellular pigment that consists of oxidized or otherwise damaged proteins, lipids (especially polyunsaturated fatty acids), and metals. As lipofuscin accumulates in many tissues with age and age-related degenerative disease, it is often considered a biomarker of aging [105–107]. Consistent with the observed increases in metals and oxidation of polyunsaturated fatty acids during senescence, senescent cells accumulate lipofuscin, which can be further visualized with Sudan Black B [108]. Lipofuscin is thought to arise from the failed degradation of macromolecules by the lysosome, resulting in persistent autofluorescent lysosomal storage bodies [109], consistent with alterations in lysosomal function during senescence. Indeed, lipofuscin is known to drive both lysosomal permeability and inflammasome activation [110], suggesting the possibility that this pigment might drive these phenotypes in senescent cells. Importantly, lipofuscin is not exclusive to senescent cells, appearing in postmitotic neurons, cardiomyocytes, and skeletal muscle cells [106, 111, 112]. Overall, lipofuscin is a useful marker of senescent cells, but like other markers it cannot be exclusively used in their identification.

## Persistent cell survival

Senescence is a stress or damage response, but it is not the only response to stress or damage. Notably, many drivers of senescence can also result in cell death modalities such as apoptosis, necroptosis, ferroptosis, and parthanatos [113]. Indeed, genetic interventions and molecules that convert senescence into cell death, often called “senolytics” regardless of cell death modality, are the primary mechanism by which senescent cells have been shown to drive degenerative pathologies [22, 114, 115]. In agreement, many of the key features of senescence, including persistent DNA damage, mitochondrial dysfunction, and oncogene activation, can also result in variegated forms of cell death. It is therefore often stated that a key feature of senescent cells is cell death resistance, but as with other features of senescence, there is considerable heterogeneity in these responses. These include:

### Apoptosis

The most-studied variant of cell death, apoptosis results in cell death via a highly regulated program that includes caspase activation, release of cytochrome C from mitochondria, DNA fragmentation and laddering, membrane phospholipid inversion, and release of intracellular contents in membrane-bound apoptotic bodies. As mentioned above, senescent cells show some features of apoptosis, such as minority mitochondrial outer membrane permeabilization, but survive in the face of these stressors [58]. This is at least in part due to elevation in anti-apoptotic proteins BCL-2, BCL-W, BCL-xL, and/or MCL1, as inhibition of these proteins selectively drives senescent cells into apoptosis [116–119], and similar results are seen with several intrinsic apoptosis inducers [120]. Apoptosis can also be triggered via the extrinsic pathway in response to cell death ligand binding, and senescent cells often elevate or secrete decoy receptors for many of these extrinsic apoptotic signaling pathways, potentially protecting them from extrinsic induction of cell death. However, senescence-inducing stimuli have also been reported to elevate the proapoptotic DR5 receptor [121, 122], though varying reports suggest both elevation or downregulation of cFLIP, depending on cell type and inducer [122–124]. This variability is seen in senescent cancer cells, where chemotherapeutic- induced senescence can confer either resistance or sensitization to TRAIL-induced apoptosis [121]. Thus, it does not appear that resistance to apoptosis is a universal feature of senescence, but rather a highly contextual phenotype.

### Lytic programmed cell death

Many forms of programmed cell death result in lysis of the cell via disruption of plasma membrane. These modalities are often highly inflammatory due to release of cellular contents, including immunogenic damage-associated molecular patterns such as nucleic acids and HMGB1 [113] – which might manifest in a manner similar to senescence at the level of tissue and biofluids. Notable modalities of lytic cell death with known relationships to senescence include necroptosis, ferroptosis, and parthanatos. Like senescence, necroptosis increases with age, in response to many of the same stimuli [125, 126]. In addition, senolytic treatment reduces markers of necroptosis, whereas inhibition of necroptosis lowers senescence, suggesting that these two processes may undergo reciprocal control [127]. Activation of necroptosis occurs primarily in response to TNF-α, a component of the SASP, suggesting that this relationship may be cell non-autonomous (i.e., senescent and necroptotic cells are distinct populations), though these links are difficult to distinguish in population/whole tissue analyses. Future studies will require specific staining of senescence and necroptosis markers in the same tissue to determine if senescent and necroptotic cells represent the same or distinct populations. Ferroptosis is another form of lytic death that results from peroxidation of the inner leaflet of cellular membranes, catalyzed by intracellular labile iron. Senescent cells display several features in common with ferroptosis, including increases in lipoxygenases [70, 71, 128], increased labile iron [102, 103, 129], and increased membrane-bound PUFA [129]. The net effect of these changes is increased sub-ferroptotic stress, similar to apoptosis. As with apoptosis, reports have indicated that senescent cells are either resistant, or sensitive, to ferroptosis [62, 100, 103]. Importantly, because senescent cells are often hypertrophic relative to non-senescent cells of the same type in culture, seeding cells at similar numbers can result in higher confluence and cell-to-cell contacts in senescent cultures relative to non-senescent. This increased confluence might explain reports of ferroptosis resistance, as cell contacts antagonize ferroptosis [130]. Finally, parthanatos is a form of lytic cell death that occurs in response to overproduction of poly-ADP-ribose from NAD+ as a result of poly-ADP-ribose polymerase (PARP) activation following genotoxic or oxidative stress [131]. It has features in common with apoptosis, such as chromatin compaction, but is lytic in that it is membrane-disruptive [131]. PARP inhibitors can prevent the development of parthanatos in response to genotoxic stress from oxidative injury, such as from ischemia-reperfusion injury, instead favoring senescence [132].

Because of these relationships and the continued survival of senescent cells, it is often stated that a key feature of cellular senescence is cell death resistance. Given the variability in these responses, it might be helpful to characterize this phenotype as persistent cell survival in the presence of cell death stress due to compensatory activation of pro-survival pathways. Because these pro-survival pathways can be sufficient or insufficient to confer resistance to exogenous death inducers depending on the cell type and inducer, contextual interpretation may be required when studying the relationship between senescence and cell death.

## CONCLUSIONS

The field of cellular senescence has come a long way since its inception, moving beyond the culture dish, into model organisms and even human patients over the course of the last 30 years. These advances have allowed for targeting senescent cells and the SASP, but the variability and non-specificity of individual senescence features necessitate robust characterization to avoid experimental artifacts. Indeed, markers of senescence are often not binary, but rather occur across a gradient [133]. Because no single feature or marker of senescent cells is exclusive to the senescent state, it is recommended that multiple markers be used together for assessment of senescence. Several reviews have proposed a consensus for a minimum set of biomarkers to confirm senescence, and the reader is directed to these for further reading [16, 17].

Conversely, cells with features of senescence can still drive pathology, even if not all criteria for characterization of senescence are met, so therapeutic opportunities may arise out of the study of senescence biology even in the absence of identification of cells with minimal requirements for senescence. For example, release from atazanavir treatment not only reduces markers of senescence in culture and *in vivo*, but also improves cardiovascular phenotype. Similarly, myofibroblasts have features in common with senescent cells and can be killed in a manner similar to senescent cells by the senolytic ABT-263, and thus improve dermal fibrosis [134].

The overlap between senescence phenotypes and macrophages raise an important question: how much of the myriad phenotypes associated with aging, regeneration, and degenerative disease that result from interventions targeted to senescence are confounded by the shared phenotypes between senescence and activated macrophages or related cells? Targeted elimination of p16-positive cells or using beta-galactosidase-targeted prodrugs is likely to eliminate at least some macrophages, and more recent studies have shown that the popular senolytic therapy of dasatinib plus quercetin can also kill p16-positive macrophages [135], suggesting that elimination of a subset of macrophages may be beneficial for age-related diseases. Interactions between senescent cells, the SASP, and macrophages add complexity to this question, and macrophages themselves may become senescent [136, 137]. Regardless of whether they are senescent or not, if macrophages that display senescence signatures turn out to be therapeutically useful targets, then the senescence features we use to identify them could be just as important as the senescent cells themselves.

As highlighted throughout this review, the senescent phenotype is complex, with variation in virtually every feature outlined in this article (Figure 1). This heterogeneity has created new translational challenges, not only in creating consensus on the identification and measurement of senescence, but also because different senescent cell populations often respond differently to intervention depending on cell type, tissue, time, and stressor [14, 104, 122, 138]. Adding to the complexity, the advent of newer technologies, from fluorescent probes to single cell and positional multi-omics, has resulted in application of these technologies to senescence [139–142]. Integration of these datasets into the larger framework of senescence biology is an evolving process that requires increasing sophistication in its analysis. This has resulted in significant development of novel computational approaches to identify and characterize senescent cells [143–150]. These approaches are likely to become more common as additional large datasets become available, but limitations remain. For example, 3’-RNA-seq often cannot distinguish between transcripts in the *CDKN1A*, *CDKN2A*, and *CDKN2B* loci, limiting the utility of retroactive analysis of datasets generated in this manner. To address these concerns, advances in transcriptomics have led to the development of several gene expression signatures used to detect and quantify senescence burden in place of the CDKNs [17, 151–153]. While these methods have limitations, they can supplement other data and allow for retroactive approximation of senescence in datasets that might not otherwise be achievable. Overall, the increasing complexity found in the features of senescence is being met with increased sophistication in their analysis, and while limitations will always exist, the ultimate goal of developing better therapies will almost certainly benefit from these approaches and a nuanced understanding of senescence in the human body.

**Figure 1:**
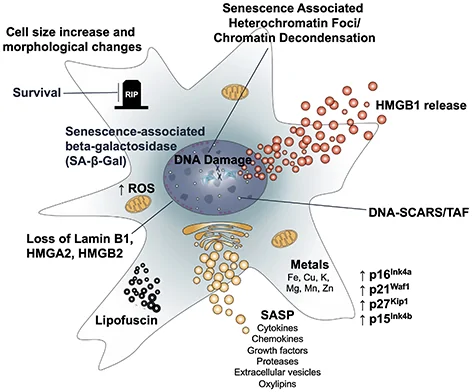
**Features of cellular senescence.** Senescence is accompanied by multiple features that are both functionally important and can also be used as biomarkers. These include (clockwise): 1. Senescence-associated beta-galactosidase and other lysosomal glycosidases. 2. Persistent cell survival*.* 3. Increased cell size and a flattened morphology. 4. Epigenetic changes including heterochromatin foci, chromatin decondensation, release of HMGB1, and persistent DNA damage foci (DNA-SCARS), including telomere-associated foci (TAF). 5. Increased cyclin-dependent kinase inhibitors, such as p16, p21, p27, or p15. 6. Accumulation of metals. 7. The SASP, consisting of hundreds of secreted molecules that include inflammatory cytokines and chemokines, growth factors, proteases, extracellular vesicles, and oxylipins. 8. Accumulation of lipofuscin. 9. Nuclear changes, including loss of Lamin B1 and HMGB2. 10. Mitochondrial expansion and dysfunction, including increased mitochondrial mass, ROS, and release of mitochondrial DNA into the cytosol.

## References

[1] Carrel A, Ebeling AH. AGE AND MULTIPLICATION OF FIBROBLASTS. J Exp Med. 1921; 34:599–623. 10.1084/jem.34.6.599 19868581 PMC2128071

[2] Witkowski JA. Dr. Carrel's immortal cells. Med Hist. 1980; 24:129–42. 10.1017/s0025727300040126 6990125 PMC1082700

[3] Hayflick L. The illusion of cell immortality. Br J Cancer. 2000; 83:841–6. 10.1054/bjoc.2000.1296 10970682 PMC2374692

[4] Hayflick L, Moorhead PS. The serial cultivation of human diploid cell strains. Exp Cell Res. 1961; 25:585–621. 10.1016/0014-4827(61)90192-6 13905658

[5] Hayflick L. THE LIMITED *IN VITRO* LIFETIME OF HUMAN DIPLOID CELL STRAINS. Exp Cell Res. 1965; 37:614–36. 10.1016/0014-4827(65)90211-9 14315085

[6] Dimri GP, Lee X, Basile G, Acosta M, Scott G, Roskelley C, Medrano EE, Linskens M, Rubelj I, Pereira-Smith O. A biomarker that identifies senescent human cells in culture and in aging skin *in vivo*. Proc Natl Acad Sci USA. 1995; 92:9363–7. 10.1073/pnas.92.20.9363 7568133 PMC40985

[7] Bodnar AG, Ouellette M, Frolkis M, Holt SE, Chiu CP, Morin GB, Harley CB, Shay JW, Lichtsteiner S, Wright WE. Extension of life-span by introduction of telomerase into normal human cells. Science. 1998; 279:349–52. 10.1126/science.279.5349.349 9454332

[8] d'Adda di Fagagna F. Living on a break: cellular senescence as a DNA-damage response. Nat Rev Cancer. 2008; 8:512–22. 10.1038/nrc2440 18574463

[9] Serrano M, Lin AW, McCurrach ME, Beach D, Lowe SW. Oncogenic ras provokes premature cell senescence associated with accumulation of p53 and p16INK4a. Cell. 1997; 88:593–602. 10.1016/s0092-8674(00)81902-9 9054499

[10] Chen Q, Ames BN. Senescence-like growth arrest induced by hydrogen peroxide in human diploid fibroblast F65 cells. Proc Natl Acad Sci USA. 1994; 91:4130–4. 10.1073/pnas.91.10.4130 8183882 PMC43738

[11] Wiley CD, Velarde MC, Lecot P, Liu S, Sarnoski EA, Freund A, Shirakawa K, Lim HW, Davis SS, Ramanathan A, Gerencser AA, Verdin E, Campisi J. Mitochondrial Dysfunction Induces Senescence with a Distinct Secretory Phenotype. Cell Metab. 2016; 23:303–14. 10.1016/j.cmet.2015.11.011 26686024 PMC4749409

[12] Efeyan A, Ortega-Molina A, Velasco-Miguel S, Herranz D, Vassilev LT, Serrano M. Induction of p53-dependent senescence by the MDM2 antagonist nutlin-3a in mouse cells of fibroblast origin. Cancer Res. 2007; 67:7350–7. 10.1158/0008-5472.CAN-07-0200 17671205

[13] Wang B, Varela-Eirin M, Brandenburg SM, Hernandez-Segura A, van Vliet T, Jongbloed EM, Wilting SM, Ohtani N, Jager A, Demaria M. Pharmacological CDK4/6 inhibition reveals a p53-dependent senescent state with restricted toxicity. EMBO J. 2022; 41:e108946. 10.15252/embj.2021108946 34985783 PMC8922251

[14] Wang C, Vegna S, Jin H, Benedict B, Lieftink C, Ramirez C, de Oliveira RL, Morris B, Gadiot J, Wang W, du Chatinier A, Wang L, Gao D, et al. Inducing and exploiting vulnerabilities for the treatment of liver cancer. Nature. 2019; 574:268–72. 10.1038/s41586-019-1607-3 31578521 PMC6858884

[15] Neurohr GE, Terry RL, Lengefeld J, Bonney M, Brittingham GP, Moretto F, Miettinen TP, Vaites LP, Soares LM, Paulo JA, Harper JW, Buratowski S, Manalis S, et al. Excessive Cell Growth Causes Cytoplasm Dilution And Contributes to Senescence. Cell. 2019; 176:1083–97.e18. 10.1016/j.cell.2019.01.018 30739799 PMC6386581

[16] Ogrodnik M, Carlos Acosta J, Adams PD, d'Adda di Fagagna F, Baker DJ, Bishop CL, Chandra T, Collado M, Gil J, Gorgoulis V, Gruber F, Hara E, Jansen-Dürr P, et al. Guidelines for minimal information on cellular senescence experimentation *in vivo*. Cell. 2024; 187:4150–75. 10.1016/j.cell.2024.05.059 39121846 PMC11790242

[17] Gorgoulis V, Adams PD, Alimonti A, Bennett DC, Bischof O, Bishop C, Campisi J, Collado M, Evangelou K, Ferbeyre G, Gil J, Hara E, Krizhanovsky V, et al. Cellular Senescence: Defining a Path Forward. Cell. 2019; 179:813–27. 10.1016/j.cell.2019.10.005 31675495

[18] López-Domínguez JA, Rodríguez-López S, Ahumada-Castro U, Desprez PY, Konovalenko M, Laberge RM, Cárdenas C, Villalba JM, Campisi J. Cdkn1a transcript variant 2 is a marker of aging and cellular senescence. Aging (Albany NY). 2021; 13:13380–92. 10.18632/aging.203110 34035185 PMC8202863

[19] Wang F, Zhang L. p15(INK4b) regulates cell cycle signaling in hippocampal astrocytes of aged rats. Aging Clin Exp Res. 2016; 28:813–21. 10.1007/s40520-015-0484-3 26526028

[20] Menthena A, Koehler CI, Sandhu JS, Yovchev MI, Hurston E, Shafritz DA, Oertel M. Activin A, p15INK4b signaling, and cell competition promote stem/progenitor cell repopulation of livers in aging rats. Gastroenterology. 2011; 140:1009–20. 10.1053/j.gastro.2010.12.003 21147108 PMC3087123

[21] Chkhotua AB, Gabusi E, Altimari A, D'Errico A, Yakubovich M, Vienken J, Stefoni S, Chieco P, Yussim A, Grigioni WF. Increased expression of p16(INK4a) and p27(Kip1) cyclin-dependent kinase inhibitor genes in aging human kidney and chronic allograft nephropathy. Am J Kidney Dis. 2003; 41:1303–13. 10.1016/s0272-6386(03)00363-9 12776284

[22] Baker DJ, Childs BG, Durik M, Wijers ME, Sieben CJ, Zhong J, Saltness RA, Jeganathan KB, Verzosa GC, Pezeshki A, Khazaie K, Miller JD, van Deursen JM. Naturally occurring p16(Ink4a)-positive cells shorten healthy lifespan. Nature. 2016; 530:184–9. 10.1038/nature16932 26840489 PMC4845101

[23] Demaria M, Ohtani N, Youssef SA, Rodier F, Toussaint W, Mitchell JR, Laberge RM, Vijg J, Van Steeg H, Dollé ME, Hoeijmakers JH, de Bruin A, Hara E, Campisi J. An essential role for senescent cells in optimal wound healing through secretion of PDGF-AA. Dev Cell. 2014; 31:722–33. 10.1016/j.devcel.2014.11.012 25499914 PMC4349629

[24] Wang B, Wang L, Gasek NS, Kuo CL, Nie J, Kim T, Yan P, Zhu J, Torrance BL, Zhou Y, Flores LC, Allen C, Andrade AM, et al. Intermittent clearance of p21-highly-expressing cells extends lifespan and confers sustained benefits to health and physical function. Cell Metab. 2024; 36:1795–805.e6. 10.1016/j.cmet.2024.07.006 39111286 PMC11315361

[25] Hall BM, Balan V, Gleiberman AS, Strom E, Krasnov P, Virtuoso LP, Rydkina E, Vujcic S, Balan K, Gitlin I, Leonova K, Polinsky A, Chernova OB, Gudkov AV. Aging of mice is associated with p16(Ink4a)- and β-galactosidase-positive macrophage accumulation that can be induced in young mice by senescent cells. Aging (Albany NY). 2016; 8:1294–315. 10.18632/aging.100991 27391570 PMC4993332

[26] Hall BM, Balan V, Gleiberman AS, Strom E, Krasnov P, Virtuoso LP, Rydkina E, Vujcic S, Balan K, Gitlin II, Leonova KI, Consiglio CR, Gollnick SO, et al. p16(Ink4a) and senescence-associated β-galactosidase can be induced in macrophages as part of a reversible response to physiological stimuli. Aging (Albany NY). 2017; 9:1867–84. 10.18632/aging.101268 28768895 PMC5611982

[27] Liu JY, Souroullas GP, Diekman BO, Krishnamurthy J, Hall BM, Sorrentino JA, Parker JS, Sessions GA, Gudkov AV, Sharpless NE. Cells exhibiting strong *p16^INK4a^* promoter activation *in vivo* display features of senescence. Proc Natl Acad Sci U S A. 2019; 116:2603–11. 10.1073/pnas.1818313116 30683717 PMC6377452

[28] Li Y, Nichols MA, Shay JW, Xiong Y. Transcriptional repression of the D-type cyclin-dependent kinase inhibitor p16 by the retinoblastoma susceptibility gene product pRb. Cancer Res. 1994; 54:6078–82. 7954450

[29] Coller HA, Sang L, Roberts JM. A new description of cellular quiescence. PLoS Biol. 2006; 4:e83. 10.1371/journal.pbio.0040083 16509772 PMC1393757

[30] Ko RM, Kim HG, Wolff L, Klug CA. Roles of p15Ink4b and p16Ink4a in myeloid differentiation and RUNX1-ETO-associated acute myeloid leukemia. Leuk Res. 2008; 32:1101–11. 10.1016/j.leukres.2007.10.012 18037485 PMC2430055

[31] Humeniuk R, Rosu-Myles M, Fares J, Koller R, Bies J, Wolff L. The role of tumor suppressor p15Ink4b in the regulation of hematopoietic progenitor cell fate. Blood Cancer J. 2013; 3:e99. 10.1038/bcj.2012.44 23359317 PMC3556574

[32] Hsu CH, Altschuler SJ, Wu LF. Patterns of Early p21 Dynamics Determine Proliferation-Senescence Cell Fate after Chemotherapy. Cell. 2019; 178:361–73.e12. 10.1016/j.cell.2019.05.041 31204100 PMC6688629

[33] Marescal O, Cheeseman IM. Cellular Mechanisms and Regulation of Quiescence. Dev Cell. 2020; 55:259–71. 10.1016/j.devcel.2020.09.029 33171109 PMC7665062

[34] Dirac AM, Bernards R. Reversal of senescence in mouse fibroblasts through lentiviral suppression of p53. J Biol Chem. 2003; 278:11731–4. 10.1074/jbc.C300023200 12551891

[35] Beauséjour CM, Krtolica A, Galimi F, Narita M, Lowe SW, Yaswen P, Campisi J. Reversal of human cellular senescence: roles of the p53 and p16 pathways. EMBO J. 2003; 22:4212–22. 10.1093/emboj/cdg417 12912919 PMC175806

[36] Wiley CD, Schaum N, Alimirah F, Lopez-Dominguez JA, Orjalo AV, Scott G, Desprez PY, Benz C, Davalos AR, Campisi J. Small-molecule MDM2 antagonists attenuate the senescence-associated secretory phenotype. Sci Rep. 2018; 8:2410. 10.1038/s41598-018-20000-4 29402901 PMC5799282

[37] Kuehnemann C, Hughes JB, Desprez PY, Melov S, Wiley CD, Campisi J. Antiretroviral protease inhibitors induce features of cellular senescence that are reversible upon drug removal. Aging Cell. 2023; 22:e13750. 10.1111/acel.13750 36539941 PMC9835573

[38] Lee S, Schmitt CA. The dynamic nature of senescence in cancer. Nat Cell Biol. 2019; 21:94–101. 10.1038/s41556-018-0249-2 30602768

[39] Martínez-Zamudio RI, Stefa A, Nabuco Leva Ferreira Freitas JA, Vasilopoulos T, Simpson M, Doré G, Roux PF, Galan MA, Chokshi RJ, Bischof O, Herbig U. Escape from oncogene-induced senescence is controlled by POU2F2 and memorized by chromatin scars. Cell Genom. 2023; 3:100293. 10.1016/j.xgen.2023.100293 37082139 PMC10112333

[40] Fleury H, Malaquin N, Tu V, Gilbert S, Martinez A, Olivier MA, Sauriol SA, Communal L, Leclerc-Desaulniers K, Carmona E, Provencher D, Mes-Masson AM, Rodier F. Exploiting interconnected synthetic lethal interactions between PARP inhibition and cancer cell reversible senescence. Nat Commun. 2019; 10:2556. 10.1038/s41467-019-10460-1 31186408 PMC6560032

[41] Milanovic M, Fan DN, Belenki D, Däbritz JH, Zhao Z, Yu Y, Dörr JR, Dimitrova L, Lenze D, Monteiro Barbosa IA, Mendoza-Parra MA, Kanashova T, Metzner M, et al. Senescence-associated reprogramming promotes cancer stemness. Nature. 2018; 553:96–100. 10.1038/nature25167 29258294

[42] Zampetidis CP, Galanos P, Angelopoulou A, Zhu Y, Polyzou A, Karamitros T, Kotsinas A, Lagopati N, Mourkioti I, Mirzazadeh R, Polyzos A, Garnerone S, Mizi A, et al. A recurrent chromosomal inversion suffices for driving escape from oncogene-induced senescence via subTAD reorganization. Mol Cell. 2021; 81:4907–23.e8. 10.1016/j.molcel.2021.10.017 34793711

[43] Evangelou K, Belogiannis K, Papaspyropoulos A, Petty R, Gorgoulis VG. Escape from senescence: molecular basis and therapeutic ramifications. J Pathol. 2023; 260:649–65. 10.1002/path.6164 37550877

[44] Lee BY, Han JA, Im JS, Morrone A, Johung K, Goodwin EC, Kleijer WJ, DiMaio D, Hwang ES. Senescence-associated beta-galactosidase is lysosomal beta-galactosidase. Aging Cell. 2006; 5:187–95. 10.1111/j.1474-9726.2006.00199.x 16626397

[45] Sun J, Wang M, Zhong Y, Ma X, Sun S, Xu C, Peng L, Li G, Zhang L, Liu Z, Ai D, Liu B. A Glb1-2A-mCherry reporter monitors systemic aging and predicts lifespan in middle-aged mice. Nat Commun. 2022; 13:7028. 10.1038/s41467-022-34801-9 36396643 PMC9671911

[46] Hildebrand DG, Lehle S, Borst A, Haferkamp S, Essmann F, Schulze-Osthoff K. α-Fucosidase as a novel convenient biomarker for cellular senescence. Cell Cycle. 2013; 12:1922–7. 10.4161/cc.24944 23673343 PMC3735706

[47] Casella G, Munk R, Kim KM, Piao Y, De S, Abdelmohsen K, Gorospe M. Transcriptome signature of cellular senescence. Nucleic Acids Res. 2019; 47:11476. 10.1093/nar/gkz879 31612919 PMC6868356

[48] Johmura Y, Yamanaka T, Omori S, Wang TW, Sugiura Y, Matsumoto M, Suzuki N, Kumamoto S, Yamaguchi K, Hatakeyama S, Takami T, Yamaguchi R, Shimizu E, et al. Senolysis by glutaminolysis inhibition ameliorates various age-associated disorders. Science. 2021; 371:265–70. 10.1126/science.abb5916 33446552

[49] Campisi J. Replicative senescence: an old lives' tale? Cell. 1996; 84:497–500. 10.1016/s0092-8674(00)81023-5 8598035

[50] Shay JW, Wright WE. Hayflick, his limit, and cellular ageing. Nat Rev Mol Cell Biol. 2000; 1:72–6. 10.1038/35036093 11413492

[51] Krtolica A, Parrinello S, Lockett S, Desprez PY, Campisi J. Senescent fibroblasts promote epithelial cell growth and tumorigenesis: a link between cancer and aging. Proc Natl Acad Sci USA. 2001; 98:12072–7. 10.1073/pnas.211053698 11593017 PMC59769

[52] Coppé JP, Patil CK, Rodier F, Sun Y, Muñoz DP, Goldstein J, Nelson PS, Desprez PY, Campisi J. Senescence-associated secretory phenotypes reveal cell-nonautonomous functions of oncogenic RAS and the p53 tumor suppressor. PLoS Biol. 2008; 6:2853–68. 10.1371/journal.pbio.0060301 19053174 PMC2592359

[53] Acosta JC, O'Loghlen A, Banito A, Guijarro MV, Augert A, Raguz S, Fumagalli M, Da Costa M, Brown C, Popov N, Takatsu Y, Melamed J, d'Adda di Fagagna F, et al. Chemokine signaling via the CXCR2 receptor reinforces senescence. Cell. 2008; 133:1006–18. 10.1016/j.cell.2008.03.038 18555777

[54] Kuilman T, Michaloglou C, Vredeveld LC, Douma S, van Doorn R, Desmet CJ, Aarden LA, Mooi WJ, Peeper DS. Oncogene-induced senescence relayed by an interleukin-dependent inflammatory network. Cell. 2008; 133:1019–31. 10.1016/j.cell.2008.03.039 18555778

[55] Freund A, Patil CK, Campisi J. p38MAPK is a novel DNA damage response-independent regulator of the senescence-associated secretory phenotype. EMBO J. 2011; 30:1536–48. 10.1038/emboj.2011.69 21399611 PMC3102277

[56] De Cecco M, Ito T, Petrashen AP, Elias AE, Skvir NJ, Criscione SW, Caligiana A, Brocculi G, Adney EM, Boeke JD, Le O, Beauséjour C, Ambati J, et al. L1 drives IFN in senescent cells and promotes age-associated inflammation. Nature. 2019; 566:73–8. 10.1038/s41586-018-0784-9 30728521 Erratum in: Nature. 2019; 572:E5. DOI: 10.1038/s41586-019-1350-9 PMID: 31296937 PMC6519963

[57] Dou Z, Ghosh K, Vizioli MG, Zhu J, Sen P, Wangensteen KJ, Simithy J, Lan Y, Lin Y, Zhou Z, Capell BC, Xu C, Xu M, et al. Cytoplasmic chromatin triggers inflammation in senescence and cancer. Nature. 2017; 550:402–6. 10.1038/nature24050 28976970 PMC5850938

[58] Victorelli S, Salmonowicz H, Chapman J, Martini H, Vizioli MG, Riley JS, Cloix C, Hall-Younger E, Machado Espindola-Netto J, Jurk D, Lagnado AB, Sales Gomez L, Farr JN, et al. Apoptotic stress causes mtDNA release during senescence and drives the SASP. Nature. 2023; 622:627–36. 10.1038/s41586-023-06621-4 37821702 PMC10584674

[59] Franceschi C, Campisi J. Chronic inflammation (inflammaging) and its potential contribution to age-associated diseases. J Gerontol A Biol Sci Med Sci. 2014; 69:S4–9. 10.1093/gerona/glu057 24833586

[60] Basisty N, Kale A, Jeon OH, Kuehnemann C, Payne T, Rao C, Holtz A, Shah S, Sharma V, Ferrucci L, Campisi J, Schilling B. A proteomic atlas of senescence-associated secretomes for aging biomarker development. PLoS Biol. 2020; 18:e3000599. 10.1371/journal.pbio.3000599 31945054 PMC6964821

[61] Schafer MJ, White TA, Iijima K, Haak AJ, Ligresti G, Atkinson EJ, Oberg AL, Birch J, Salmonowicz H, Zhu Y, Mazula DL, Brooks RW, Fuhrmann-Stroissnigg H, et al. Cellular senescence mediates fibrotic pulmonary disease. Nat Commun. 2017; 8:14532. 10.1038/ncomms14532 28230051 PMC5331226

[62] Wiley CD, Liu S, Limbad C, Zawadzka AM, Beck J, Demaria M, Artwood R, Alimirah F, Lopez-Dominguez JA, Kuehnemann C, Danielson SR, Basisty N, Kasler HG, et al. SILAC Analysis Reveals Increased Secretion of Hemostasis-Related Factors by Senescent Cells. Cell Rep. 2019; 28:3329–37.e5. 10.1016/j.celrep.2019.08.049 31553904 PMC6907691

[63] Meng Q, Chen C, Yang N, Gololobova O, Shi C, Dunn CA, Rossi M, Martindale JL, Basisty N, Ding J, Delannoy M, Basu S, Mazan-Mamczarz K, et al. Surfaceome analysis of extracellular vesicles from senescent cells uncovers uptake repressor DPP4. Proc Natl Acad Sci USA. 2023; 120:e2219801120. 10.1073/pnas.2219801120 37862381 PMC10614838

[64] Jeon OH, Wilson DR, Clement CC, Rathod S, Cherry C, Powell B, Lee Z, Khalil AM, Green JJ, Campisi J, Santambrogio L, Witwer KW, Elisseeff JH. Senescence cell-associated extracellular vesicles serve as osteoarthritis disease and therapeutic markers. JCI Insight. 2019; 4:e125019. 10.1172/jci.insight.125019 30944259 PMC6483636

[65] Takasugi M, Okada R, Takahashi A, Virya Chen D, Watanabe S, Hara E. Small extracellular vesicles secreted from senescent cells promote cancer cell proliferation through EphA2. Nat Commun. 2017; 8:15729. 10.1038/ncomms15728 28585531 PMC5467215

[66] Borghesan M, Fafián-Labora J, Eleftheriadou O, Carpintero-Fernández P, Paez-Ribes M, Vizcay-Barrena G, Swisa A, Kolodkin-Gal D, Ximénez-Embún P, Lowe R, Martín-Martín B, Peinado H, Muñoz J, et al. Small Extracellular Vesicles Are Key Regulators of Non-cell Autonomous Intercellular Communication in Senescence via the Interferon Protein IFITM3. Cell Rep. 2019; 27:3956–71.e6. 10.1016/j.celrep.2019.05.095 31242426 PMC6613042

[67] Patel SK, Bons J, Rose JP, Chappel JR, Beres RL, Watson MA, Webster C, Burton JB, Bruderer R, Desprez PY, Reiter L, Campisi J, Baker ES, Schilling B. Exosomes released from senescent cells and circulatory exosomes isolated from human plasma reveal aging-associated proteomic and lipid signatures. Aging (Albany NY). 2025; 17:1929–65. 10.18632/aging.206292 40748658 PMC12422824

[68] Li J, Tan J, Song Q, Yang X, Zhang X, Qin H, Huang G, Su X, Li J. Exosomal miR-767 from senescent endothelial-derived accelerating skin fibroblasts aging via inhibiting TAB1. J Mol Histol. 2023; 54:13–24. 10.1007/s10735-022-10107-4 36409439 PMC9908644

[69] Lee AH, Ghosh D, Koh IL, Dawson MR. Senescence-associated exosomes transfer miRNA-induced fibrosis to neighboring cells. Aging (Albany NY). 2023; 15:1237–56. 10.18632/aging.204539 36842089 PMC10042705

[70] Wiley CD, Sharma R, Davis SS, Lopez-Dominguez JA, Mitchell KP, Wiley S, Alimirah F, Kim DE, Payne T, Rosko A, Aimontche E, Deshpande SM, Neri F, et al. Oxylipin biosynthesis reinforces cellular senescence and allows detection of senolysis. Cell Metab. 2021; 33:1124–36.e5. 10.1016/j.cmet.2021.03.008 33811820 PMC8501892

[71] Wiley CD, Brumwell AN, Davis SS, Jackson JR, Valdovinos A, Calhoun C, Alimirah F, Castellanos CA, Ruan R, Wei Y, Chapman HA, Ramanathan A, Campisi J, Jourdan Le Saux C. Secretion of leukotrienes by senescent lung fibroblasts promotes pulmonary fibrosis. JCI Insight. 2019; 4:e130056. 10.1172/jci.insight.130056 31687975 PMC6975274

[72] Davalos AR, Kawahara M, Malhotra GK, Schaum N, Huang J, Ved U, Beausejour CM, Coppe JP, Rodier F, Campisi J. p53-dependent release of Alarmin HMGB1 is a central mediator of senescent phenotypes. J Cell Biol. 2013; 201:613–29. 10.1083/jcb.201206006 23649808 PMC3653366

[73] Chen R, Kang R, Tang D. The mechanism of HMGB1 secretion and release. Exp Mol Med. 2022; 54:91–102. 10.1038/s12276-022-00736-w 35217834 PMC8894452

[74] Parry AJ, Hoare M, Bihary D, Hänsel-Hertsch R, Smith S, Tomimatsu K, Mannion E, Smith A, D'Santos P, Russell IA, Balasubramanian S, Kimura H, Samarajiwa SA, Narita M. NOTCH-mediated non-cell autonomous regulation of chromatin structure during senescence. Nat Commun. 2018; 9:1840. 10.1038/s41467-018-04283-9 29743479 PMC5943456

[75] Acosta JC, Banito A, Wuestefeld T, Georgilis A, Janich P, Morton JP, Athineos D, Kang TW, Lasitschka F, Andrulis M, Pascual G, Morris KJ, Khan S, et al. A complex secretory program orchestrated by the inflammasome controls paracrine senescence. Nat Cell Biol. 2013; 15:978–90. 10.1038/ncb2784 23770676 PMC3732483

[76] Rovira M, Sereda R, Pladevall-Morera D, Ramponi V, Marin I, Maus M, Madrigal-Matute J, Díaz A, García F, Muñoz J, Cuervo AM, Serrano M. The lysosomal proteome of senescent cells contributes to the senescence secretome. Aging Cell. 2022; 21:e13707. 10.1111/acel.13707 36087066 PMC9577959

[77] Tao W, Yu Z, Han JJ. Single-cell senescence identification reveals senescence heterogeneity, trajectory, and modulators. Cell Metab. 2024; 36:1126–43.e5. 10.1016/j.cmet.2024.03.009 38604170

[78] Cohn RL, Gasek NS, Kuchel GA, Xu M. The heterogeneity of cellular senescence: insights at the single-cell level. Trends Cell Biol. 2023; 33:9–17. 10.1016/j.tcb.2022.04.011 35599179 PMC9812642

[79] Uyar B, Palmer D, Kowald A, Murua Escobar H, Barrantes I, Möller S, Akalin A, Fuellen G. Single-cell analyses of aging, inflammation and senescence. Ageing Res Rev. 2020; 64:101156. 10.1016/j.arr.2020.101156 32949770 PMC7493798

[80] Hernandez-Segura A, de Jong TV, Melov S, Guryev V, Campisi J, Demaria M. Unmasking Transcriptional Heterogeneity in Senescent Cells. Curr Biol. 2017; 27:2652–60.e4. 10.1016/j.cub.2017.07.033 28844647 PMC5788810

[81] Freund A, Laberge RM, Demaria M, Campisi J. Lamin B1 loss is a senescence-associated biomarker. Mol Biol Cell. 2012; 23:2066–75. 10.1091/mbc.E11-10-0884 22496421 PMC3364172

[82] Gasior SL, Wakeman TP, Xu B, Deininger PL. The human LINE-1 retrotransposon creates DNA double-strand breaks. J Mol Biol. 2006; 357:1383–93. 10.1016/j.jmb.2006.01.089 16490214 PMC4136747

[83] Aird KM, Iwasaki O, Kossenkov AV, Tanizawa H, Fatkhutdinov N, Bitler BG, Le L, Alicea G, Yang TL, Johnson FB, Noma KI, Zhang R. HMGB2 orchestrates the chromatin landscape of senescence-associated secretory phenotype gene loci. J Cell Biol. 2016; 215:325–34. 10.1083/jcb.201608026 27799366 PMC5100296

[84] Rodier F, Coppé JP, Patil CK, Hoeijmakers WA, Muñoz DP, Raza SR, Freund A, Campeau E, Davalos AR, Campisi J. Persistent DNA damage signalling triggers senescence-associated inflammatory cytokine secretion. Nat Cell Biol. 2009; 11:973–9. 10.1038/ncb1909 19597488 PMC2743561

[85] Rodier F, Muñoz DP, Teachenor R, Chu V, Le O, Bhaumik D, Coppé JP, Campeau E, Beauséjour CM, Kim SH, Davalos AR, Campisi J. DNA-SCARS: distinct nuclear structures that sustain damage-induced senescence growth arrest and inflammatory cytokine secretion. J Cell Sci. 2011; 124:68–81. 10.1242/jcs.071340 21118958 PMC3001408

[86] Hewitt G, Jurk D, Marques FD, Correia-Melo C, Hardy T, Gackowska A, Anderson R, Taschuk M, Mann J, Passos JF. Telomeres are favoured targets of a persistent DNA damage response in ageing and stress-induced senescence. Nat Commun. 2012; 3:708. 10.1038/ncomms1708 22426229 PMC3292717

[87] Fumagalli M, Rossiello F, Clerici M, Barozzi S, Cittaro D, Kaplunov JM, Bucci G, Dobreva M, Matti V, Beausejour CM, Herbig U, Longhese MP, d'Adda di Fagagna F. Telomeric DNA damage is irreparable and causes persistent DNA-damage-response activation. Nat Cell Biol. 2012; 14:355–65. 10.1038/ncb2466 Erratum in: Nat Cell Biol. 2012; 14:555. DOI: 10.1038/ncb2466 PMID:22426077 PMC3717580 22426077

[88] Narita M, Nũnez S, Heard E, Narita M, Lin AW, Hearn SA, Spector DL, Hannon GJ, Lowe SW. Rb-mediated heterochromatin formation and silencing of E2F target genes during cellular senescence. Cell. 2003; 113:703–16. 10.1016/s0092-8674(03)00401-x 12809602

[89] Swanson EC, Manning B, Zhang H, Lawrence JB. Higher-order unfolding of satellite heterochromatin is a consistent and early event in cell senescence. J Cell Biol. 2013; 203:929–42. 10.1083/jcb.201306073 24344186 PMC3871423

[90] Ziegler DV, Wiley CD, Velarde MC. Mitochondrial effectors of cellular senescence: beyond the free radical theory of aging. Aging Cell. 2015; 14:1–7. 10.1111/acel.12287 25399755 PMC4310776

[91] Miwa S, Kashyap S, Chini E, von Zglinicki T. Mitochondrial dysfunction in cell senescence and aging. J Clin Invest. 2022; 132:e158447. 10.1172/JCI158447 35775483 PMC9246372

[92] Wiley CD, Campisi J. The metabolic roots of senescence: mechanisms and opportunities for intervention. Nat Metab. 2021; 3:1290–301. 10.1038/s42255-021-00483-8 34663974 PMC8889622

[93] Roh K, Noh J, Kim Y, Jang Y, Kim J, Choi H, Lee Y, Ji M, Kang D, Kim MS, Paik MJ, Chung J, Kim JH, et al. Lysosomal control of senescence and inflammation through cholesterol partitioning. Nat Metab. 2023; 5:398–413. 10.1038/s42255-023-00747-5 36864206

[94] Ivanov A, Pawlikowski J, Manoharan I, van Tuyn J, Nelson DM, Rai TS, Shah PP, Hewitt G, Korolchuk VI, Passos JF, Wu H, Berger SL, Adams PD. Lysosome-mediated processing of chromatin in senescence. J Cell Biol. 2013; 202:129–43. 10.1083/jcb.201212110 23816621 PMC3704985

[95] Tan JX, Finkel T. Lysosomes in senescence and aging. EMBO Rep. 2023; 24:e57265. 10.15252/embr.202357265 37811693 PMC10626421

[96] Kang HT, Lee KB, Kim SY, Choi HR, Park SC. Autophagy impairment induces premature senescence in primary human fibroblasts. PLoS One. 2011; 6:e23367. 10.1371/journal.pone.0023367 21858089 PMC3152578

[97] Wang H, Muthu Karuppan MK, Devadoss D, Nair M, Chand HS, Lakshmana MK. TFEB protein expression is reduced in aged brains and its overexpression mitigates senescence-associated biomarkers and memory deficits in mice. Neurobiol Aging. 2021; 106:26–36. 10.1016/j.neurobiolaging.2021.06.003 34229273

[98] Killilea DW, Atamna H, Liao C, Ames BN. Iron accumulation during cellular senescence in human fibroblasts *in vitro*. Antioxid Redox Signal. 2003; 5:507–16. 10.1089/152308603770310158 14580305 PMC4503765

[99] Killilea DW, Wong SL, Cahaya HS, Atamna H, Ames BN. Iron accumulation during cellular senescence. Ann N Y Acad Sci. 2004; 1019:365–7. 10.1196/annals.1297.063 15247045

[100] Masaldan S, Clatworthy SA, Gamell C, Meggyesy PM, Rigopoulos AT, Haupt S, Haupt Y, Denoyer D, Adlard PA, Bush AI, Cater MA. Iron accumulation in senescent cells is coupled with impaired ferritinophagy and inhibition of ferroptosis. Redox Biol. 2018; 14:100–15. 10.1016/j.redox.2017.08.015 28888202 PMC5596264

[101] Masaldan S, Clatworthy SAS, Gamell C, Smith ZM, Francis PS, Denoyer D, Meggyesy PM, Fontaine S, Cater MA. Copper accumulation in senescent cells: Interplay between copper transporters and impaired autophagy. Redox Biol. 2018; 16:322–31. 10.1016/j.redox.2018.03.007 29579719 PMC5953000

[102] Maus M, López-Polo V, Mateo L, Lafarga M, Aguilera M, De Lama E, Meyer K, Sola A, Lopez-Martinez C, López-Alonso I, Guasch-Piqueras M, Hernandez-Gonzalez F, Chaib S, et al. Iron accumulation drives fibrosis, senescence and the senescence-associated secretory phenotype. Nat Metab. 2023; 5:2111–30. 10.1038/s42255-023-00928-2 38097808 PMC10730403

[103] Admasu TD, Kim K, Rae M, Avelar R, Gonciarz RL, Rebbaa A, Pedro de Magalhães J, Renslo AR, Stolzing A, Sharma A. Selective ablation of primary and paracrine senescent cells by targeting iron dyshomeostasis. Cell Rep. 2023; 42:112058. 10.1016/j.celrep.2023.112058 36753419

[104] Du K, Umbaugh DS, Wang L, Jun JH, Dutta RK, Oh SH, Ren N, Zhang Q, Ko DC, Ferreira A, Hill J, Gao G, Pullen SS, et al. Targeting senescent hepatocytes for treatment of metabolic dysfunction-associated steatotic liver disease and multi-organ dysfunction. Nat Commun. 2025; 16:3038. 10.1038/s41467-025-57616-w 40155379 PMC11953480

[105] Terman A, Brunk UT. Lipofuscin. Int J Biochem Cell Biol. 2004; 36:1400–4. 10.1016/j.biocel.2003.08.009 15147719

[106] Gray DA, Woulfe J. Lipofuscin and aging: a matter of toxic waste. Sci Aging Knowledge Environ. 2005; 2005:re1. 10.1126/sageke.2005.5.re1 15689603

[107] Terman A, Brunk UT. Lipofuscin: mechanisms of formation and increase with age. APMIS. 1998; 106:265–76. 10.1111/j.1699-0463.1998.tb01346.x 9531959

[108] Georgakopoulou EA, Tsimaratou K, Evangelou K, Fernandez Marcos PJ, Zoumpourlis V, Trougakos IP, Kletsas D, Bartek J, Serrano M, Gorgoulis VG. Specific lipofuscin staining as a novel biomarker to detect replicative and stress-induced senescence. A method applicable in cryo-preserved and archival tissues. Aging (Albany NY). 2013; 5:37–50. 10.18632/aging.100527 23449538 PMC3616230

[109] Katz ML, Robison WG Jr. What is lipofuscin? Defining characteristics and differentiation from other autofluorescent lysosomal storage bodies. Arch Gerontol Geriatr. 2002; 34:169–84. 10.1016/s0167-4943(02)00005-5 14764321

[110] Pan C, Banerjee K, Lehmann GL, Almeida D, Hajjar KA, Benedicto I, Jiang Z, Radu RA, Thompson DH, Rodriguez-Boulan E, Nociari MM. Lipofuscin causes atypical necroptosis through lysosomal membrane permeabilization. Proc Natl Acad Sci U S A. 2021; 118:e2100122118. 10.1073/pnas.2100122118 34782457 PMC8617501

[111] Kakimoto Y, Okada C, Kawabe N, Sasaki A, Tsukamoto H, Nagao R, Osawa M. Myocardial lipofuscin accumulation in ageing and sudden cardiac death. Sci Rep. 2019; 9:3304. 10.1038/s41598-019-40250-0 30824797 PMC6397159

[112] Lu JQ, Monaco CM, Hawke TJ, Yan C, Tarnopolsky MA. Increased intra-mitochondrial lipofuscin aggregates with spherical dense body formation in mitochondrial myopathy. J Neurol Sci. 2020; 413:116816. 10.1016/j.jns.2020.116816 32272361

[113] Galluzzi L, Vitale I, Warren S, Adjemian S, Agostinis P, Martinez AB, Chan TA, Coukos G, Demaria S, Deutsch E, Draganov D, Edelson RL, Formenti SC, et al. Consensus guidelines for the definition, detection and interpretation of immunogenic cell death. J Immunother Cancer. 2020; 8:e000337. 10.1136/jitc-2019-000337 32209603 PMC7064135

[114] Xu M, Pirtskhalava T, Farr JN, Weigand BM, Palmer AK, Weivoda MM, Inman CL, Ogrodnik MB, Hachfeld CM, Fraser DG, Onken JL, Johnson KO, Verzosa GC, et al. Senolytics improve physical function and increase lifespan in old age. Nat Med. 2018; 24:1246–56. 10.1038/s41591-018-0092-9 29988130 PMC6082705

[115] Baar MP, Brandt RMC, Putavet DA, Klein JDD, Derks KWJ, Bourgeois BRM, Stryeck S, Rijksen Y, van Willigenburg H, Feijtel DA, van der Pluijm I, Essers J, van Cappellen WA, et al. Targeted Apoptosis of Senescent Cells Restores Tissue Homeostasis in Response to Chemotoxicity and Aging. Cell. 2017; 169:132–47.e16. 10.1016/j.cell.2017.02.031 28340339 PMC5556182

[116] Chang J, Wang Y, Shao L, Laberge RM, Demaria M, Campisi J, Janakiraman K, Sharpless NE, Ding S, Feng W, Luo Y, Wang X, Aykin-Burns N, et al. Clearance of senescent cells by ABT263 rejuvenates aged hematopoietic stem cells in mice. Nat Med. 2016; 22:78–83. 10.1038/nm.4010 26657143 PMC4762215

[117] Rysanek D, Vasicova P, Kolla JN, Sedlak D, Andera L, Bartek J, Hodny Z. Synergism of BCL-2 family inhibitors facilitates selective elimination of senescent cells. Aging (Albany NY). 2022; 14:6381–414. 10.18632/aging.204207 35951353 PMC9467395

[118] Yosef R, Pilpel N, Tokarsky-Amiel R, Biran A, Ovadya Y, Cohen S, Vadai E, Dassa L, Shahar E, Condiotti R, Ben-Porath I, Krizhanovsky V. Directed elimination of senescent cells by inhibition of BCL-W and BCL-XL. Nat Commun. 2016; 7:11190. 10.1038/ncomms11190 27048913 PMC4823827

[119] Zhu Y, Tchkonia T, Fuhrmann-Stroissnigg H, Dai HM, Ling YY, Stout MB, Pirtskhalava T, Giorgadze N, Johnson KO, Giles CB, Wren JD, Niedernhofer LJ, Robbins PD, et al. Identification of a novel senolytic agent, navitoclax, targeting the Bcl-2 family of anti-apoptotic factors. Aging Cell. 2016; 15:428–35. 10.1111/acel.12445 26711051 PMC4854923

[120] Tepper CG, Seldin MF, Mudryj M. Fas-mediated apoptosis of proliferating, transiently growth-arrested, and senescent normal human fibroblasts. Exp Cell Res. 2000; 260:9–19. 10.1006/excr.2000.4990 11010806

[121] Soto-Gamez A, Wang Y, Zhou X, Seras L, Quax W, Demaria M. Enhanced extrinsic apoptosis of therapy-induced senescent cancer cells using a death receptor 5 (DR5) selective agonist. Cancer Lett. 2022; 525:67–75. 10.1016/j.canlet.2021.10.038 34728311

[122] Wang L, Jin H, Jochems F, Wang S, Lieftink C, Martinez IM, De Conti G, Edwards F, de Oliveira RL, Schepers A, Zhou Y, Zheng J, Wu W, et al. cFLIP suppression and DR5 activation sensitize senescent cancer cells to senolysis. Nat Cancer. 2022; 3:1284–99. 10.1038/s43018-022-00462-2 36414711

[123] Stagni V, Mingardi M, Santini S, Giaccari D, Barilà D. ATM kinase activity modulates cFLIP protein levels: potential interplay between DNA damage signalling and TRAIL-induced apoptosis. Carcinogenesis. 2010; 31:1956–63. 10.1093/carcin/bgq193 20876284

[124] Belharazem D, Grass A, Paul C, Vitacolonna M, Schalke B, Rieker RJ, Körner D, Jungebluth P, Simon-Keller K, Hohenberger P, Roessner EM, Wiebe K, Gräter T, et al. Increased cFLIP expression in thymic epithelial tumors blocks autophagy via NF-κB signalling. Oncotarget. 2017; 8:89580–94. 10.18632/oncotarget.15929 29163772 PMC5685693

[125] Deepa SS, Unnikrishnan A, Matyi S, Hadad N, Richardson A. Necroptosis increases with age and is reduced by dietary restriction. Aging Cell. 2018; 17:e12770. 10.1111/acel.12770 29696779 PMC6052392

[126] Royce GH, Brown-Borg HM, Deepa SS. The potential role of necroptosis in inflammaging and aging. Geroscience. 2019; 41:795–811. 10.1007/s11357-019-00131-w 31721033 PMC6925091

[127] Thadathil N, Selvarani R, Mohammed S, Nicklas EH, Tran AL, Kamal M, Luo W, Brown JL, Lawrence MM, Borowik AK, Miller BF, Van Remmen H, Richardson A, Deepa SS. Senolytic treatment reduces cell senescence and necroptosis in Sod1 knockout mice that is associated with reduced inflammation and hepatocellular carcinoma. Aging Cell. 2022; 21:e13676. 10.1111/acel.13676 35869934 PMC9381894

[128] Catalano A, Rodilossi S, Caprari P, Coppola V, Procopio A. 5-Lipoxygenase regulates senescence-like growth arrest by promoting ROS-dependent p53 activation. EMBO J. 2005; 24:170–9. 10.1038/sj.emboj.7600502 15616590 PMC544914

[129] Kuehnemann C, Jezak S, Mogck B, Kura N, Che Y, Vaittinen M, Siokas I, Tariq R, Deshpande S, Guthrie C, Tan X, Aimontche E, Zheng T, et al. Fatty acid desaturation guides cellular decisions between ferroptosis and cellular senescence. bioRxiv. 2024.10.03.616257. 10.1101/2024.10.03.616257

[130] Wu J, Minikes AM, Gao M, Bian H, Li Y, Stockwell BR, Chen ZN, Jiang X. Intercellular interaction dictates cancer cell ferroptosis via NF2-YAP signalling. Nature. 2019; 572:402–6. 10.1038/s41586-019-1426-6 31341276 PMC6697195

[131] Fatokun AA, Dawson VL, Dawson TM. Parthanatos: mitochondrial-linked mechanisms and therapeutic opportunities. Br J Pharmacol. 2014; 171:2000–16. 10.1111/bph.12416 24684389 PMC3976618

[132] Nehme J, Mesilmany L, Varela-Eirin M, Brandenburg S, Altulea A, Lin Y, Gaya da Costa M, Seelen M, Hillebrands JL, van Goor H, Saab R, Akl H, Prevarskaya N, et al. Converting cell death into senescence by PARP1 inhibition improves recovery from acute oxidative injury. Nat Aging. 2024; 4:771–82. 10.1038/s43587-024-00627-x 38724734

[133] Ashraf HM, Fernandez B, Spencer SL. The intensities of canonical senescence biomarkers integrate the duration of cell-cycle withdrawal. Nat Commun. 2023; 14:4527. 10.1038/s41467-023-40132-0 37500655 PMC10374620

[134] Lagares D, Santos A, Grasberger PE, Liu F, Probst CK, Rahimi RA, Sakai N, Kuehl T, Ryan J, Bhola P, Montero J, Kapoor M, Baron M, et al. Targeted apoptosis of myofibroblasts with the BH3 mimetic ABT-263 reverses established fibrosis. Sci Transl Med. 2017; 9:eaal3765. 10.1126/scitranslmed.aal3765 29237758 PMC8520471

[135] Grosse L, Wagner N, Emelyanov A, Molina C, Lacas-Gervais S, Wagner KD, Bulavin DV. Defined p16^High^ Senescent Cell Types Are Indispensable for Mouse Healthspan. Cell Metab. 2020 Jul 7; 32:87–99.e6. 10.1016/j.cmet.2020.05.002 32485135

[136] Behmoaras J, Gil J. Similarities and interplay between senescent cells and macrophages. J Cell Biol. 2021; 220:e202010162. 10.1083/jcb.202010162 33355620 PMC7769159

[137] Elder SS, Emmerson E. Senescent cells and macrophages: key players for regeneration? Open Biol. 2020; 10:200309. 10.1098/rsob.200309 33352064 PMC7776574

[138] Zhu Y, Tchkonia T, Pirtskhalava T, Gower AC, Ding H, Giorgadze N, Palmer AK, Ikeno Y, Hubbard GB, Lenburg M, O'Hara SP, LaRusso NF, Miller JD, et al. The Achilles' heel of senescent cells: from transcriptome to senolytic drugs. Aging Cell. 2015; 14:644–58. 10.1111/acel.12344 25754370 PMC4531078

[139] Neri F, Basisty N, Desprez PY, Campisi J, Schilling B. Quantitative Proteomic Analysis of the Senescence-Associated Secretory Phenotype by Data-Independent Acquisition. Curr Protoc. 2021; 1:e32. 10.1002/cpz1.32 33524224 PMC7898702

[140] Nguyen ND, Rosas L, Khaliullin T, Jiang P, Hasanaj E, Ovando-Ricardez JA, Bueno M, Rahman I, Pryhuber GS, Li D, Ma Q, Finkel T, Königshoff M, et al. scDOT: optimal transport for mapping senescent cells in spatial transcriptomics. Genome Biol. 2024; 25:288. 10.1186/s13059-024-03426-0 39516853 PMC11546560

[141] Wang Z, Ge S, Liao T, Yuan M, Qian W, Chen Q, Liang W, Cheng X, Zhou Q, Ju Z, Zhu H, Xiong W. Integrative single-cell metabolomics and phenotypic profiling reveals metabolic heterogeneity of cellular oxidation and senescence. Nat Commun. 2025; 16:2740. 10.1038/s41467-025-57992-3 40113759 PMC11926267

[142] Suryadevara V, Hudgins AD, Rajesh A, Pappalardo A, Karpova A, Dey AK, Hertzel A, Agudelo A, Rocha A, Soygur B, Schilling B, Carver CM, Aguayo-Mazzucato C, et al. SenNet recommendations for detecting senescent cells in different tissues. Nat Rev Mol Cell Biol. 2024; 25:1001–23. 10.1038/s41580-024-00738-8 38831121 PMC11578798

[143] Duran I, Pombo J, Sun B, Gallage S, Kudo H, McHugh D, Bousset L, Barragan Avila JE, Forlano R, Manousou P, Heikenwalder M, Withers DJ, Vernia S, et al. Detection of senescence using machine learning algorithms based on nuclear features. Nat Commun. 2024; 15:1041. 10.1038/s41467-024-45421-w 38310113 PMC10838307

[144] Mahmud S, Zheng C, Santiago FE, Zhang L, Robbins PD, Dong X. A machine learning approach identifies cellular senescence on transcriptome data of human cells *in vitro*. Geroscience. 2025; 47:5287–301. 10.1007/s11357-024-01485-6 39738795 PMC12181610

[145] Hughes BK, Wallis R, Bishop CL. Yearning for machine learning: applications for the classification and characterisation of senescence. Cell Tissue Res. 2023; 394:1–16. 10.1007/s00441-023-03768-4 37016180 PMC10558380

[146] Mapkar SA, Bliss SA, Perez Carbajal EE, Murray SH, Li Z, Wilson AK, Piprode V, Lee YJ, Kirsch T, Petroff KS, Liu F, Wosczyna MN. Nuclear morphometrics coupled with machine learning identifies dynamic states of senescence across age. Nat Commun. 2025; 16:6231. 10.1038/s41467-025-60975-z 40624008 PMC12234852

[147] Qu Y, Ji B, Dong R, Gu L, Chan C, Xie J, Glass C, Wang XF, Nixon AB, Ji Z. Single-cell and spatial detection of senescent cells using DeepScence. Cell Genom. 2025; 5:101035. 10.1016/j.xgen.2025.101035 41061702 PMC12802646

[148] Sanborn MA, Wang X, Gao S, Dai Y, Rehman J. Unveiling the cell-type-specific landscape of cellular senescence through single-cell transcriptomics using SenePy. Nat Commun. 2025; 16:1884. 10.1038/s41467-025-57047-7 39987255 PMC11846890

[149] Avelar RA, Ortega JG, Tacutu R, Tyler EJ, Bennett D, Binetti P, Budovsky A, Chatsirisupachai K, Johnson E, Murray A, Shields S, Tejada-Martinez D, Thornton D, et al. A multidimensional systems biology analysis of cellular senescence in aging and disease. Genome Biol. 2020; 21:91. 10.1186/s13059-020-01990-9 32264951 PMC7333371

[150] Fernandez B, Passanisi V, Ashraf HM, Spencer SL. Single-cell RNA sequencing reveals distinct senotypes and a quiescence-senescence continuum at the transcriptome level following chemotherapy. bioRxiv. 2025; 17:169. 10.1038/s41467-025-66836-z 41436452 PMC12780064

[151] Jochems F, Thijssen B, De Conti G, Jansen R, Pogacar Z, Groot K, Wang L, Schepers A, Wang C, Jin H, Beijersbergen RL, Leite de Oliveira R, Wessels LF, Bernards R. The Cancer SENESCopedia: A delineation of cancer cell senescence. Cell Rep. 2021; 36:109441. 10.1016/j.celrep.2021.109441 34320349 PMC8333195

[152] Saul D, Kosinsky RL, Atkinson EJ, Doolittle ML, Zhang X, LeBrasseur NK, Pignolo RJ, Robbins PD, Niedernhofer LJ, Ikeno Y, Jurk D, Passos JF, Hickson LJ, et al. A new gene set identifies senescent cells and predicts senescence-associated pathways across tissues. Nat Commun. 2022; 13:4827. 10.1038/s41467-022-32552-1 35974106 PMC9381717

[153] Hughes BK, Davis A, Milligan D, Wallis R, Mossa F, Philpott MP, Wainwright LJ, Gunn DA, Bishop CL. SenPred: a single-cell RNA sequencing-based machine learning pipeline to classify deeply senescent dermal fibroblast cells for the detection of an *in vivo* senescent cell burden. Genome Med. 2025; 17:2. 10.1186/s13073-024-01418-0 39810225 PMC11731430

